# Prophylactic Neuroprotection of Total Glucosides of Paeoniae Radix Alba against Semen Strychni-Induced Neurotoxicity in Rats: Suppressing Oxidative Stress and Reducing the Absorption of Toxic Components

**DOI:** 10.3390/nu10040514

**Published:** 2018-04-20

**Authors:** Shujuan Li, Yanjie Chu, Ruowen Zhang, Linjia Sun, Xiaohui Chen

**Affiliations:** 1 School of Pharmacy, Shenyang Pharmaceutical University, Shenyang Pharmaceutical University, No. 103, Wenhua Road, Shenyang 110000, China; shujuanli@hotmail.com (S.L.); sunlj1995@hotmail.com (L.S.); 2 School of Traditional Chinese Material Medica, Shenyang Pharmaceutical University, No. 103, Wenhua Road, Shenyang 110000, China; cyj15566235674@hotmail.com; 3 Southern Research Institute, 2000 9th Ave. s., Birmingham 35205, AL, USA; zrw_shrimp@hotmail.com

**Keywords:** Semen Strychni, neurotoxicity, Paeoniae Radix Alba, neuro-protective effects

## Abstract

Strychnos alkaloids (SAs) are the main toxic constituents in Semen Strychni, a traditional Chinese medicine, which is known for its fatal neurotoxicity. Hence, the present study was carried out to evaluate the neurotoxicity induced by SAs and the pre-protective effects of the total glucosides of Paeoniae Radix Alba (TGP). An SA brain damage model was firstly established. The neurotoxicity induced by SAs and the pre-protective effects of TGP were confirmed by physical and behavioral testing, biochemical assay, and histological examination. Then, a liquid chromatography-tandem mass spectrometry method was developed and validated to investigate the time-course change and distribution of strychnine and brucine (two main SAs) in the brain after oral SA administration with or without TGP pretreatment. Biochemical analysis results indicated that TGP could ameliorate the oxidative stress status caused by SAs. Time-course change and distribution studies demonstrated that strychnine and brucine were rapidly absorbed into the brain, peaked early at 0.5 h, and were mainly located in the hippocampus and cerebellum. TGP showed a pre-protective effect against neurotoxicity by reducing the absorption of toxic alkaloids into the brain. These findings could provide beneficial information in facilitating future studies of Semen Strychni neurotoxicity and developing herbal medicines to alleviate neurotoxicity in the clinic.

## 1. Introduction

Semen Strychni, the mature seed from *Strychnos nux-vomica* L., has been used as a traditional Chinese medicine (TCM) for hundreds of years, and is listed in Chinese pharmacopeia for the treatment of facial nerve paralysis, rheumatoid arthritis, and bone fractures [1]. However, due to the extreme neurotoxicity of Semen Strychni, it is strictly controlled by the government and required to be processed (such as parching in a sand bath) before use or compatibility with other remedial TCMs [2]. It is reported that Strychnos alkaloids (SAs) are responsible for the pharmacological and toxic properties of Semen Strychni [3]. To date, a total of 16 alkaloids have been documented and identified in Semen Strychni extract, among which strychnine (Str) and brucine (Bru) account for up to 70% [4].

The toxic alkaloids in Semen Strychni are lipophilic and thus SAs distribute rapidly and widely in the body and can penetrate the blood-brain barrier (BBB) [5]. Studies indicated that SAs could block postsynaptic inhibitory glycine receptors in the spinal cord and brain stem, which may contribute to one of the Semen Strychni-induced neurotoxicity mechanisms [2,6]. Due to the narrow margin between effective and toxic doses, neurotoxicity induced by SAs is likely to occur with exposure to Semen Strychni. The results in our previous study showed that continuous administration of SAs could induce neurotoxicity. The main toxic effects manifested changes in rat behavior and disorders of endogenous components [7,8].

The protective properties of herbal medicines have attracted increasing attention. Various crude extracts or drugs, such as ginsenosides and berberine, derived from TCMs play a key role in the treatment of neurological diseases [9,10]. Total glucosides of Paeoniae Radix Alba (TGP) are a class of active compounds extracted from the roots of *Paeonia lactiflora* Pall., which has long been used in TCMs to alleviate various disorders [11,12,13]. Recent studies demonstrated that TGP, especially paeoniflorin (the most abundant ingredient), have been widely used in the treatment of central nervous system (CNS) diseases [14,15]. The neuroprotective potential of TGP may be related to mediation of inflammatory responses, oxidative stress, and endogenous metabolic processes both in vivo [7,16] and in vitro [17,18].

In recent decades, many studies have investigated the metabolic [19,20] and pharmacokinetic [21,22] behavior of SAs in blood. Few studies have examined the profile of SAs in the brain after oral administration. The quantitation of SAs in the brain is crucial for their potential to induce neurotoxic effects. Furthermore, the brain levels of SAs are likely to be more accurate to evaluate neurotoxic effects on the CNS compared to plasma levels of SAs, especially in the case of pre-protection or compatibility with other TCMs to alleviate SAs-induced toxicity. After SAs are transported through the BBB and are efficiently delivered to the brain, they require appropriate distribution and concentration at considerable levels in the target regions. SAs can then be related to neurotoxicity. Hence, studying the dynamic change and distribution of toxic components is necessary and essential, as this can determine the possible toxic targets and help to reveal the mechanism of action. However, there are no reports on the distribution of SAs in rat brain. Thus, the objective of the present study was to establish a new method to simultaneously quantitate and explore the distribution of the two main SAs (Str and Bru) in the brain using a high-performance liquid chromatography-tandem mass spectrometer (HPLC-MS/MS) system. Meanwhile, the influence of TGP on the pharmacokinetic effects of toxic compounds from SAs was also investigated.

In this paper, an animal model of brain injury induced by SAs was established. Then, based on that animal model, the time-course change and distribution of Str and Bru in the brain were investigated with or without pretreatment of TGP. Our results will help to study the influences of TGP on the process of absorption and distribution of Str and Bru in the brain from a quantitative view. This investigation will greatly improve the understanding of the neural toxicity induced by Semen Strychni. Our findings might provide beneficial information in developing herbal medicines to alleviate neurotoxicity in the clinic.

## 2. Materials and Methods

### 2.1. Chemicals and Materials

Semen Strychni, Paeoniae Radix Alba, and Flos Daturae were obtained commercially from Anguo Lengbei Co., Ltd. (Anguo, China). Voucher specimens (Semen Strychni, MQZ-2016-002, Paeoniae Radix Alba, BS-2016-001, Flos Daturae, YJH-2016-001,) were deposited in the School of Pharmacy, Shenyang Pharmaceutical University. Standards of Str, Bru, and acetaminophen (internal standard, IS) were acquired from the National Institute for Control of Pharmaceutical and Biological Products (Beijing, China). The purity of each standard was not less than 98.5%. All commercial kits used in the experiment were purchased from Nanjing Jiancheng Biotech Inc. (Nanjing, China) including acetylcholinesterase (AChE), monoamine oxidase (MAO), Na^+^-K^+^ ATPase, superoxide dismutase (SOD), catalase (CAT), reduced glutathione (GSH), total antioxidant capacity (T-AOC), reactive oxygen species (ROS), brain-derived neurotrophic factor (BDNF), and nerve growth factor (NGF). Acetonitrile (HPLC grade) was provided by Fisher Scientific (Pittsburgh, PA, USA). Formic acid was supplied by Kermel Chemical Reagent Co., Ltd. (Tianjin, China). All primers were synthesized by Thermo Fisher Scientific (Shanghai, China). Other chemicals were of analytical grade or better unless otherwise specified.

### 2.2. Herb Preparation

The herbal extraction of Semen Strychni and Flos Daturae followed the procedure of Li et al. [20] and Shi et al. [7], respectively. Extraction protocols are described briefly as follows:

The smashed Semen Strychni was extracted by refluxing with 50% ethanol three times, 1 h each time. The collected filtrate was concentrated under reduced pressure and extracted further with methylene chloride. Extracting solvent was removed by rotary evaporator and the yield of SAs contained 35.5% Str and 24.4% Bru as determined by an HPLC method.

Raw Flos Daturae was extracted by refluxing with 75% ethanol three times, 1 h each time. The collected filtrate was concentrated under reduced pressure. The resultant residue was dried at room temperature.

A modified version of the extraction protocol described by Deng et al. [13] was applied to prepare TGP. Sliced Paeoniae Radix Alba was extracted three times with 70% ethanol (1:8, *w*/*v*) for two hours each time. The extracts were obtained by concentrating the filtrates and drying under vacuum. Then the crude sample was suspended in water and chromatographed on macroporous resins with 70% ethanol. The eluate was collected and evaporated to dryness under reduced pressure, which yielded a dry powder of TGP.

### 2.3. Animal Treatment 

The study was approved by the Institutional Animal Ethical Committee of Shenyang Pharmaceutical University (approval ID: 17403). Experimental animals (male, pathogen-free Sprague-Dawley rats, 220–250 g) were kindly supplied by the Experimental Animal Center of Shenyang Pharmaceutical University. Rats were housed in an air-conditioned animal center (humidity, 50 ± 10%; temperature, 22 ± 2 °C) with a 12 h light/dark cycle. Before the experiment, all animals were allowed ad libitum to access to drinking water and standard rat chow and provided a 5-day acclimation period. There were two main parts in this study: the establishment of a rat brain injury model caused by SAs, and a brain time-course change and distribution of Str and Bru. Accompanying the two experiments, the intervention group of TGP was established to assess the effects of TGP on neurotoxicity induced by SAs.

**Figure 1:**
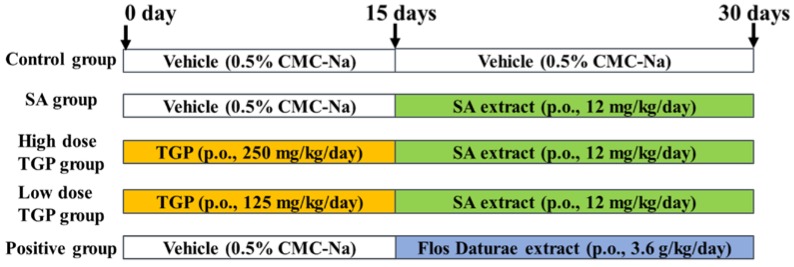
Schematic representation of the experimental procedure. SA, Strychnos alkaloid; TGP, total glucosides of Paeoniae Radix Alba. CMC, carboxyl methyl cellulose.

For the study of the establishment of rat brain injury, rats were randomly divided into five groups (*n* = 6/group): control group, SA group, low dose TGP group, high dose TGP group, and positive group. The dosing protocol is shown in Figure 1. Different doses of SAs (8, 10, 11 12, 13, 16 mg/kg) were tested in our previous study. We found that 12 mg/kg/day SAs (equivalent to 0.552 g/kg/day for Semen Strychni) was the maximum tolerated dose and could cause obvious toxic effects for rats in the experiment. Chinese pharmacopeia records the daily dose of Paeoniae Radix Alba ranging from 6 to 15 g/day for adults. The doses of TGP for rats, 125 mg/kg/day and 250 mg/kg/day, were equivalent to 6 g/day and 12 g/day for adults. A positive control group was set in the experimental design to help analyze the results of behavioral tests, biochemical assay, as well as histopathology. The dosing route and time in the positive group were the same as the SA group. Rats in the positive group were orally administered Flos Daturae extract (3.6 g/kg/day) for 15 days. Flos Daturae was selected as the positive herbal medicine due to its definite neurotoxicity [23]. Its dose schedule followed Shi et al. [7,8]. Animals were sacrificed at 0.5 h after oral administration of SA extract on the 30th day. Brains were dissected, washed with chilled saline, weighed, and divided symmetrically in half—one for the biochemical assay and the other for the histopathology study.

For the brain time-course change and distribution studies, rats were randomly divided into three groups: the control group, SA group, and TGP group (high dose). The dosing protocol was performed as above. For the brain time-course study, animals were sacrificed serially at 15-, 25-, 30-, 35-, 45-, 60-, 75-, 90-, 120-, 150-, 180-, 240-min after oral SA administration on the 30th day. There were at least three rats per time point and dose. The whole brain was obtained, washed with chilled saline, weighed, and stored at −80 °C until analysis. For the brain distribution experiment (six rats in each group), the whole brains were obtained at 30 min after SA administration on the 30th day, washed with chilled saline, and dissected to isolate cerebral cortex, hippocampus, striatum, hypothalamus, and cerebellum. The different brain regions that were collected were all stored at −80 °C until analysis.

### 2.4. Physical and Behavioral Testing

Physical and behavioral testing was performed after SA administration on the 30th day including body weight, coat state test, and beam walking test. Coat state was evaluated in various body areas of rats (the head, neck, back, tail, and paws). A score of 0 was recorded if clean, 1 if unkempt for each body area [24]. The final score was the sum of scores from five different body areas.

Motor coordination was assessed by the beam walking test. Rats had to cross a 2.5 cm-wide beam, 80 cm in length, elevated at 10 cm above the floor. The performance of rats was scored based on the following scoring system: 0 = inability to stay on the beam, 1 = stayed on the beam but inability to move, 2 = tried to traverse the beam but fell, 3 = traversed the beam with more than three slips, 4 = traversed the beam with less two slips, 5 = traversed the beam without slips [25]. Rats that achieved normal performance (a score of 5) after training were selected for the beam walking test.

### 2.5. Biochemical Assay and Histopathology

#### 2.5.1. Biochemical Assay

The brain was homogenized (10%, *w*/*v*) in ice-cold saline solution and centrifuged at 2500 r/min at 4 °C for 10 min. The supernatant was used for biochemical assay. AChE activity was assessed according to the method of Ellman et al. [26]. Thiocholine reacted with a specific chromogenic agent to produce yellow complex (Sym-trinitrobenzene). MAO activity was assayed in a manner that was dependent on the ability of the content of phenylhydrazone to be measured by a spectrophotometer in the presence of MAO and benzylamine. Na^+^-K^+^ ATPase activity was measured by detecting the release of inorganic phosphorus (Pi) from ATP catabolism. CAT activity was assayed by calculating the decomposition rate of H_2_O_2_ (ammonium molybdate method). SOD activity was determined with a hydroxylamine method. Hydroxylamine is oxidized by O^2−^ anions to form nitrites (purple in the presence of a chromogenic agent), which is markedly inhibited by SOD. The level of GSH was detected based on the reaction of dithiobisnitrobenzoic acid to form a yellow complex. T-AOC was assessed by the production of Fe^2+^, which was measured with phenanthroline colorimetry. The level of ROS was determined by a fluorescent probe (2′,7′-dichlorodihydrofluorescei diacetate). An enzyme-linked immunosorbent assay (ELISA) was applied to determine BDNF and NGF. All procedures followed the instruction manuals provided with test kits.

#### 2.5.2. Histological Examination

The brain was fixed in 10% formalin, dehydrated in ethanol, embedded in paraffin, and sliced into 5-μm thickness for staining. Brain sections were stained with hematoxylin and eosin (H&E) for morphological observation. Nile-red staining was conducted to measure the intracellular lipid accumulation according to the protocol provided with the kit (GENMED, Shanghai, China). Tissue sections were deparaffinized and rehydrated for terminal deoxynucleotidyl transferase-mediated deoxyuridine 5-triphosphate-biotin end labeling (TUNEL) staining. After permeabilization, sections were incubated with terminal deoxynucleotidyl transferase mixture for 60 min at 37 °C. To visualize the nuclei, 4′,6-diamidino-2-phenylindole (DAPI) was used for counterstaining.

### 2.6. Quantitative Real-Time Polymerase Chain Reaction (RT-PCR)

Total RNA was extracted from the brain tissues using a Trizol extraction method (Invitrogen, Carlsbad, CA, USA) according to protocols provided by the manufacturer. After RNA was evaluated by a spectrophotometer, 2 μg of RNA was reverse-transcribed into cDNA using a Prime-Script RT-PCR kit (Takara). Forward and reverse primers were 5′-TTTGTAGATGACCATGAGTCGC-3′ and 5′-TGTCCTGCTGTATGCTGCTT-3′ for nuclear factor E2-related factor 2 (Nrf2), 5′-AGCGAAACAAGCAGAACCCA-3′ and 5′-ACCTCGTGGAGACGCTTTAC-3′ for heme oxygenase (HO-1), 5′-CCAGTGCGGCCTTAGTAGAG-3′ and 5′-CCTCAGACTCCGCCTTGTTT-3′ for heat shock protein 70 (HSP-70), and 5′-TACTCTGTGTGGATTGGTGGC-3′ and 5′-GGTGTAAAACGCAGCTCAGTAA-3′ for β-actin. Amplification was performed using ABI 7500 Real-Time PCR (Applied Biosystems, USA) under the following conditions: 40 cycles at 95 °C for 10 s, 60 °C for 10 s, and 72 °C for 10 s. Relative gene expression was calculated by the 2^−ΔΔCt^.

### 2.7. HPLC-MS/MS Analysis

#### 2.7.1. Instrumentation and Operation Conditions

An ACQUITY high-performance liquid chromatography system (Waters Corp., Milford, MA, USA) interfaced to a Waters Xevo TQ mass spectrometer (Milford, MA, USA) with an electrospray ionization (ESI) was operated for LC-MS/MS analysis. MassLynx 4.1 software by Waters (Milford, MA, USA) was used for instrument control and data processing.

A CAPCELL CORE ADME column (2.1 × 150 mm, 2.7 μm, Shiseido, Tokyo, Japan) coupled with an ACQUITY BEH UPLC C-18 guard column (2.1 mm × 5 mm, Waters, USA) was used to separate the analytes. The mobile phase A was 0.1% formic acid in water, mobile phase B was acetonitrile. Analytes were separated using a gradient method, with a 0.3 mL/min flow rate, 0–2 min, 90–85% A; 2–3 min, 85–75% A; 3–6 min, 75% A; 6–7 min, 75–90% A; 7–8 min, 90% A. The injection volume was 2 μL and the autosampler injection needle was washed after each injection with methanol/water (95:5, *v*/*v*). Nitrogen, the desolvation gas, was set at a flow rate of 700 L/h. The desolvation temperature and source temperature were 400 °C and 150 °C, respectively. A multiple reaction monitoring (MRM) mode was applied for quantification of analytes. Table 1 provides the MRM settings for each compound.

#### 2.7.2. Working Solution Preparation

The IS compound was dissolved in acetonitrile/water (50:50, *v*/*v*) and diluted in acetonitrile to form a working solution at a concentration of 1.0 µg/mL. For Str and Bru, the primary stock solutions were prepared at 1.0 mg/mL in acetonitrile, individually. Working stock solutions containing both Str and Bru were diluted in acetonitrile from individual stock solutions for the preparation of calibration and quality control (QC) solutions. Calibration standard solutions were used at concentrations of 5.0, 10.0, 50.0, 100.0, 500.0, 2000.0, and 4000.0 ng/mL for Str and 1.0, 2.0, 10.0, 20.0, 100.0, 400.0, and 800.0 ng/mL for Bru. Quality control working solutions were prepared at concentrations of 10.0, 100.0, 3200.0 for Str and 2.0, 20.0, 640.0 for Bru. All solutions were stored in the refrigerator (4 °C) until use.

#### 2.7.3. Sample Preparation

One-step protein precipitation was performed to prepare samples. Frozen brains were thawed on ice before the experiment. Brain homogenate was prepared in a ratio of 3 weight units of acetonitrile and 1 weight unit of brain tissue, using a ULTRA-TURRAX homogenizer (IKA, Staufen im Breisgau, Germany), and then centrifuged at 4000 r/min for 5 min. The supernatant was obtained for further analysis. To each 500 μL supernatant, 25 μL working solution and 25 μL IS working solution (1.0 µg/mL) were added. The mixture was vortexed for 60 s and centrifuged at 12,000 r/min at 4 °C for 5 min. The supernatant was transferred and evaporated to dryness under a gentle stream of nitrogen gas at 30 °C. The remaining residue was reconstituted with 200 μL acetonitrile: water (70:30, *v*/*v*), sonicated, vortexed, and centrifuged at 16,000 r/min at 4 °C for 10 min. Two microliters (2 μL) of the supernatant was injected for analysis.

#### 2.7.4. Method Validation

To ensure the acceptability of the bioanalytical method, tests of selectivity, linearity, intra- and inter-day precision and accuracy, recovery, matrix effect, and stability were conducted according to the U.S. Food & Drug Administration (FDA) guidelines. The test operation is shown in the Supplemental Material.

### 2.8. Data Processing

Statistical data were compared using SPSS 19.0 software for Windows (SPSS Inc., Chicago, IL, USA). Differences between groups were evaluated by one-way analysis of variance (ANOVA) followed by Fisher’s least significant difference (LSD) test. Values were expressed as the mean ± standard deviation (mean ± SD). *p* < 0.05 was considered a significant difference for the test. For studies of time-course change and brain distribution, differences between the SA group and TGP group were analyzed by Tukey’s comparison test. The level of significance was *p* < 0.05.

## 3. Results

### 3.1. Physical and Behavioral Testing

With the time of SA exposure increasing, the weight of the rats decreased gradually in the SA group and the coat state of rats was in a deterioration condition in the SA group. Rats showed slight convulsions, tremors, and motor disturbance at about 30 min after SA administration in the SA group. However, convulsions or tremors were not observed in the TGP group. Compared to the control group, body weight decreased significantly in the SA group (*p* < 0.01) and positive group (*p* < 0.01, Table 2), while that in the TGP groups recovered. The degree of body weight increase in the high TGP group was more than the low TGP group. A significant deterioration of the coat state was observed in the SA group (*p* < 0.01) and positive group (*p* < 0.01, Table 2). Pretreatment of TGP at a high dose improved significantly (*p* < 0.05) the poor coat state induced by SAs. A significant decrease in the score of the beam walking test was observed in the SA group (*p* < 0.01) and positive group (*p* < 0.01, Table 2). Pretreatment with TGP, especially the high dose of TGP, prevented impairment on the motor coordination.

### 3.2. Biochemical Assay

**Figure 2:**
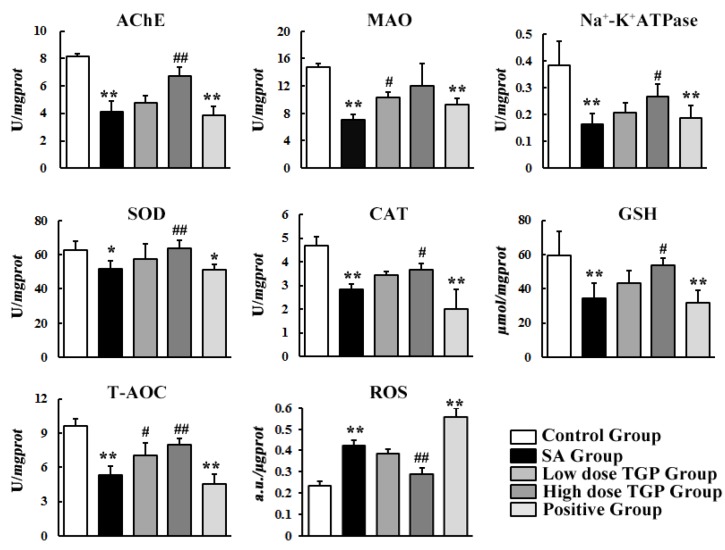
Determination results of rat brain samples collected from different groups. Each value was represented as mean ± SD (standard deviation). (* *p* < 0.05, ** *p* < 0.01 SA group vs. control group; # *p* < 0.05, ## *p* < 0.01 TGP group vs. SA group). AChE, acetylcholinesterase; MAO, monoamine oxidase; SOD, superoxide dismutase; CAT, catalase; GSH, reduced glutathione; T-AOC, total antioxidant capacity; ROS, reactive oxygen species.

Results of biochemical assay are shown in Figure 2. Compared to those in the control group, the activities of AChE, MAO, and Na^+^-K^+^ ATPase decreased significantly (*p* < 0.05) in the SA group by 50.1%, 52.5%, and 57.3%, respectively, and in the positive group by 53.1%, 37.5%, and 51.1%, respectively. Compared to those in the SA group, the activities of AChE, MAO, and Na^+^-K^+^ ATPase increased in the low TGP group by 16.5%, 47.0%, and 26.5%, respectively, and in the high dose group by 64.2%, 70.4%, and 62.1%, respectively. Pretreatment with TGP in the high dose group or low dose group resulted in increasing enzymic activity.

As shown in Figure 2, the activities of SOD and CAT and levels of reduced GSH and T-AOC decreased obviously (*p* < 0.05) in the SA group and positive group compared to the control group. However, TGP pretreatment at a low dose or high dose led to partial alleviation. The activities of SOD and CAT and levels of GSH and T-AOC increased in the low TGP group by 11.3%, 21.7%, 26.3%, and 32.3%, and in the high TGP group by 23.1%, 30.0%, 57.7%, and 48.5%, individually, compared to the SA group. The content of ROS in the SA group and positive group increased obviously by 81% and 130% compared to the control group, respectively, while the presence of TGP at a high dose caused a 46% decrease in ROS compared to the SA group. No significant difference was observed between the SA group and low TGP dose group.

### 3.3. Histopathological Examination

**Figure 3:**
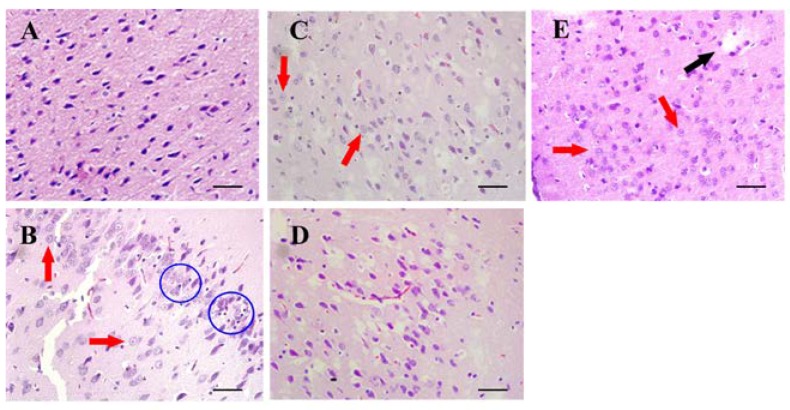
Representative histopathological photographs of rat cerebral cortexes (20×): (**A**) control group; (**B**) SA group; (**C**) low dose TGP group; (**D**) high dose TGP group; (**E**) positive group. The black arrow, liquefactive necrosis; red arrows, glial cells; blue circles, neuronophagia. Scale bar = 50 μm.

**Figure 4:**
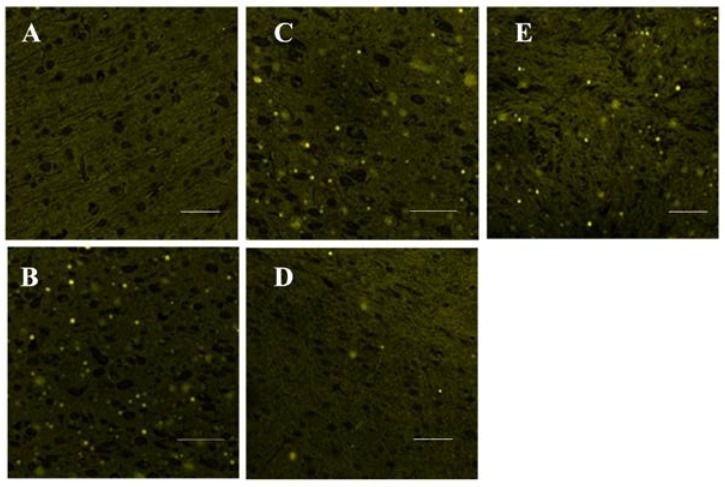
Representative photographs of Nile-red staining (400×). (**A**) control group; (**B**) SA group; (**C**) low dose TGP group; (**D**) high dose TGP group; (**E**) positive group. Scale bar = 50 μm.

**Figure 5:**
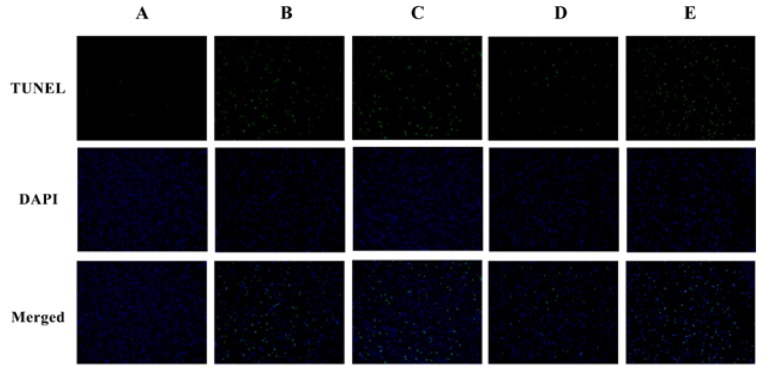
Representative photographs of terminal deoxynucleotidyl transferase-mediated deoxyuridine 5-triphosphate-biotin end labeling (TUNEL) staining (100×). (**A**) control group; (**B**) SA group; (**C**) low dose TGP group; (**D**) high dose TGP group; (**E**) positive group.

The neurotoxicity induced by SAs and the pre-protection of TGP were confirmed further by histopathological observations. Figure 3 shows representative histopathological photographs of cerebral cortexes collected from different groups. The most remarkable changes observed in the SA group and positive group were gliocyte hyperplasia. Neuronophagia was also observed in the SA group. Neutral lipid droplets were markedly increased in the SA group but not in the high dose TGP pre-treatment group revealed by Nile-red staining (Figure 4). Furthermore, oral administration of SAs could also lead to obvious cell apoptosis in the cerebral cortex (Figure 5). Histopathological examination showed that pretreatment with TGP could attenuate those morphological changes and cell apoptosis.

The above results illustrate that successive administration of SAs for 15 days could induce rat brain injury, and the pretreatment of TGP could alleviate neurotoxicity induced by SAs. In addition, it was concluded that high dose TGP had better pre-protective effects than low dose TGP, according to results from histopathological examination and biochemical assay. Therefore, the high dose of TGP (250 mg/kg/day) was chosen for the following brain time-course and distribution studies in the TGP group.

### 3.4. RT-PCR

**Figure 6:**
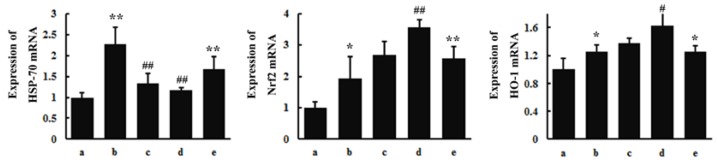
The gene expression levels of heat shock protein 70 (HSP-70), nuclear factor E2-related factor 2 (Nrf2), and heme oxygenase (HO-1) in rat brain. (**a**) control group; (**b**) SA group; (**c**) low dose TGP group; (**d**) high dose TGP group; (**e**) positive group. Values were expressed as mean ± SD. (* *p* < 0.05, ** *p* < 0.01 other groups vs. control group; # *p* < 0.05, ## *p* < 0.01 TGP group vs. SA group).

RT-PCR analysis was conducted to further evaluate the toxic effects of SAs and the pre-protection of TGP. The gene expression levels of HSP-70, Nrf2, and HO-1 in the rat brain are shown in Figure 6. SA exposure for 15 days upregulated the expression levels of HSP-70 (*p* < 0.01), Nrf2 (*p* < 0.05), and HO-1(*p* < 0.05). Similar results were also observed in the positive group when compared to the control group. Pretreatment with TGP inhibited the gene expression of HSP-70 (*p* < 0.01), while the gene expression of Nrf2 (*p* < 0.05) and HO-1 (*p* < 0.01) was induced significantly (high dose TGP group).

### 3.5. HPLC-MS/MS Method Validation

#### 3.5.1. Selectivity

**Figure 7:**
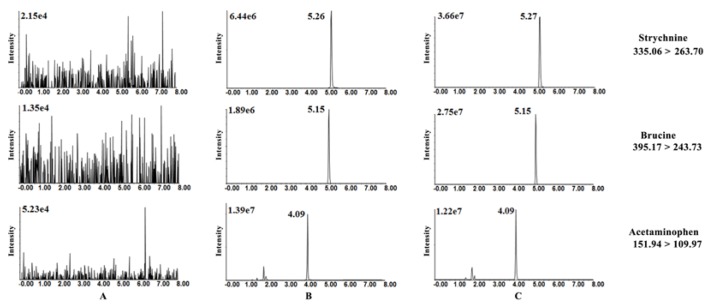
Typical multiple reaction monitoring chromatograms of each analyte and IS. (**A**) Blank brain homogenate, (**B**) spiked brain homogenate with analytes and IS (QC1), and (**C**) the brain homogenate obtained 0.5 h after oral administration.

Selectivity was assessed by comparing the chromatograms of blank brain homogenate samples, spiked brain homogenate with standards and brain homogenate obtained at 0.5 h after oral administration of SAs. No significant interference from matrix was observed (Figure 7). This demonstrated that the developed method showed good selectivity to differentiate analytes in brain homogenate.

#### 3.5.2. Linearity

Slopes, intercepts, and *r* from calibration curves are shown in Table 3. The *r* values were greater than 0.99 for each analyte, thus demonstrating good linearity of the method when determining Str and Bru in brain homogenate within the tested range.

#### 3.5.3. Precision and Accuracy

The intra-day (*n* = 6) and inter-day (*n* = 18) precision and accuracy for each analyte were all below 15% (see Supplemental Table S1) and satisfied the FDA requirements for bioanalytical method validation. In addition, we also validated the precision and accuracy of the lower limit of quantification (LLOQ) concentration in the brain homogenate for each analyte. The relative standard deviation (RSD) and relative error (RE) were all within 20% (see Supplemental Table S1). The present LC-MS/MS method met the accepted requirements of accuracy and precision.

#### 3.5.4. Recovery and Matrix Effect

The recoveries of analytes and IS were all above 89.8% at three different concentrations (see Supplemental Table S2), demonstrating that the recoveries of all analytes were consistent and reproducible. The matrix effect results (see Supplemental Table S2) were 96.5–98.1% for Str, 97.5–103.8% for Bru, and 100.4% for IS. Data indicated that no significant matrix effects were observed.

#### 3.5.5. Stability

The results demonstrated that the analytes were stable at room temperature for 8 h with RSD all less than 11.2%, under the autosampler condition (4 °C) for 12 h with RSD values all below 10.9%, three freeze-thaw cycles (RSD below 14.3%), and at −80 °C for a month (RSD below 11.2%), which validated the chemical stability of analytes in the matrix under common sample processing and storage conditions.

### 3.6. Tissue Time-Course Following Oral Administration

**Figure 8:**
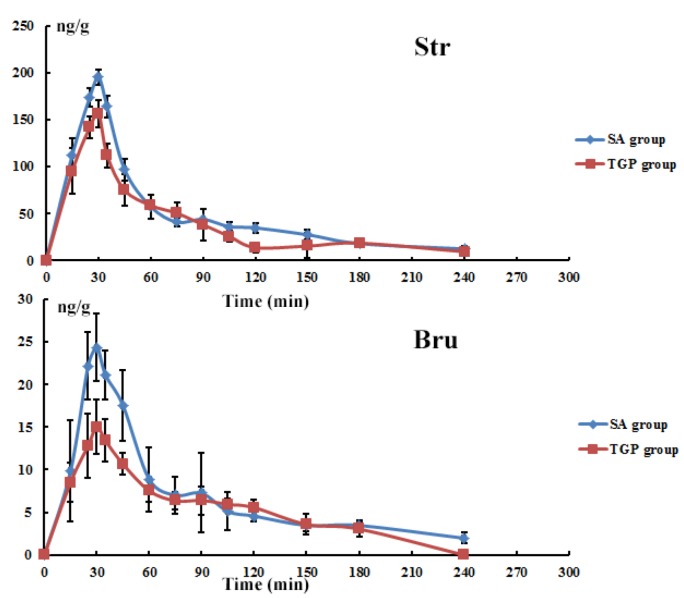
Tissue time course of Str and Bru in the SA group and TGP group in brain. There were at least three rats per time point in each group. Each point represents mean ± SD.

The absorption of Str and Bru into the brain following oral administration of SAs was rapid (detected at 15 min), peaked early (30 min), and then began to decrease over time (Figure 8). They were not detected in the brain after 240-min post administration. The absorption and elimination of Str and Bru were similar in rats with or without TGP pretreatment (250 mg/kg/day). However, a 1.25-fold and a 1.62-fold significant (*p* < 0.05) difference were observed at the maximal concentration (*C_max_*) of Str and Bru between the SA group and TGP group, respectively.

### 3.7. Brain Distribution

**Figure 9:**
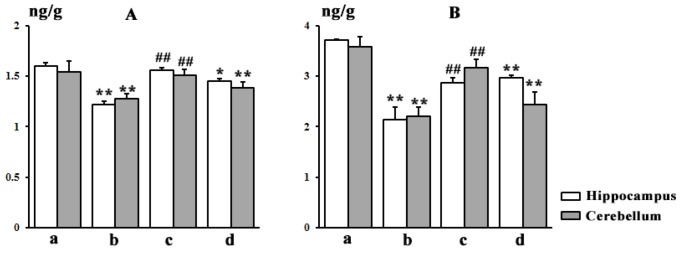
The brain-derived neurotrophic factor (BDNF) (**A**) and nerve growth factor (NGF) (**B**) levels of different group in the hippocampus and cerebellum. (a) control group; (b) SA group; (c) high dose TGP group; (d) positive group. (* *p* < 0.05, ** *p* < 0.01 other groups vs. control group; # *p* < 0.05, ## *p* < 0.01 TGP group vs. SA group).

Table 4 lists the concentrations of Str and Bru recovered from different regions. The contents of the two main SAs in different brain regions exhibited levels in the following order: hippocampus > cerebellum > striatum > hypothalamus > cerebral cortex. The concentrations of Str and Bru in the hippocampus and cerebellum were higher than in other brain regions. Significant differences (*p* < 0.05) in the contents were observed for Str in the hippocampus and cerebellum, and for Bru only in the hippocampus, between the SA group and TGP group. These findings suggested that the hippocampus and cerebellum might be targets for toxic SAs. To further verify the toxic targets of SAs, BDNF and NGF were examined. The levels of BDNF and NGF are shown in Figure 9. In the SA group, the levels of BDNF and NGF decreased significantly (*p* < 0.01) in the hippocampus and cerebellum compared to the control group. Meanwhile, TGP could cause significant increases for BDNF and NGF in the hippocampus and cerebellum, suggesting that TGP show neural pre-protective effects.

## 4. Discussion

### 4.1. Effects of TGP on SAs-Induced Neurotoxicity

Semen Strychni is classified as a highly toxic herb medicine in Chinese pharmacopeia. Oral administration of SAs for 15 days induced impairments on rats’ physical state and motor coordination in this study. Rats in the SA group had a low score for the beam walking test and showed motor disturbance. The results are consistent with previous reports that exposure to a high dose of Semen Strychni could cause motor disturbance by preventing release of the inhibitory neurotransmitter [6]. Pretreatment with TGP revealed an increase in the body weight and the score of the beam walking test. It may indicate the neural pre-protective effects of TGP against the toxicity caused by SAs preliminary.

AChE, MAO, and Na^+^-K^+^ ATPase play a vital role in the metabolism of neurotransmitters [27,28,29,30]. They can be the targets of toxic chemicals, which affect the metabolism of neurotransmitters and cause disordered nerve function by inhibiting their activities [31,32,33]. Our laboratory and collaborators found significant alterations in the contents of five neurotransmitters after continuous exposure to SA (12 mg/kg/day) [7]. According to their findings, it was deduced that SAs can have negative effects on the activities of MAO, AChE, and Na^+^-K^+^ ATPase. As expected, the results of this study indicated that oral administration of SAs for 15 days induced significant (*p* < 0.05) inhibition of MAO, AChE, and Na^+^-K^+^ ATPase activities. MAO, AChE, and Na^+^-K^+^ ATPase are usually used as biological markers of neurotoxicity [34]. Significant changes in these enzymic activities in the SA group may suggest that SAs could induce neurotoxicity. The activities of MAO and AChE were somewhat modulated by different dosages of TGP compared to the SA group. These results showed that SAs were responsible for neurotoxicity and that TGP distinguished pre-protective effects against SA toxicity.

Studies showed a close correlation between the alterations of AChE and the increased oxidative stress in both rodents and humans [35]. Since the brain is particularly sensitive to oxidative damage due to its high oxygen consumption [36], oxidative stress indexes were evaluated in this study. SOD and CAT play an important part in defense enzymes, which neutralize the common toxic effects of oxygen metabolism [37]. These two antioxidant enzymes represent the first line of defense against the toxic effects of ROS. Inhibition of CAT and SOD in the SA group caused decreased ROS consumption and led to excessive accumulation of ROS in the brain. The observed reduction in GSH and T-AOC in the SA group is likely responsible for their exhaustion in countering excessive accumulation of ROS. In parallel, TGP pretreatment led to increased activities of CAT and SOD, elevated levels of GSH and T-AOC, and decreased content of ROS, which suggested further that TGP exhibited pre-protective effects via suppression of oxidative stress and amelioration of these antioxidant systems.

Upon onset of oxidative stress in neurons, the redox balance is seriously affected, ROS are massively produced, and antioxidant enzymes are inhibited [38]. Conversely, the internal store of antioxidant GSH is severely depleted. Excessive amounts of ROS also result in lipid peroxidation and lead to lipid metabolism disorders and impaired cellular membrane function. SA exposure induced lipid accumulation and the formation of lipid droplets, and consequent cell apoptosis. High gene expression of HSP-70 indicated oxidative damage in the SA group; it may also reflect the CNS self-protection due to the anti-apoptosis characteristics of HSP-70 [39]. As a major regulator of anti-oxidative defense responses, Nrf2 is dissociated from Keap1 protein and translocated to the nucleus where it activates the antioxidant response element (ARE) under conditions of oxidative stress [40]. Here, TGP pretreatment upregulated the gene expression of Nrf2 and its target gene HO-1 as a response to oxidative damage. In addition, TGP downregulated the gene expression of HSP-70, which confirmed that TGP pretreatment likely mitigated the burden of oxidative stress.

The decrease in antioxidant abilities and enzyme activities caused by SA exposure suggested that oxidative stress might be one of the most positive neurotoxicity mechanisms of SAs. In this study, the pre-protective effects of TGP were mainly considered to decrease the excess production of ROS and improve oxidative stress status. Studies have demonstrated that *Paeonia lactiflora* extract could protect dopaminergic cells from oxidative damage via regulation of corresponding gene expression [17]. It is speculated that the pre-protective effects of TGP contribute to their immunomodulation characteristics [16] and an increasing detoxification capability of the body for toxic components. The further mechanisms by which TGP show pre-protective effects on oxidative stress caused by SAs will require deeper studies.

### 4.2. Method Development

The validated method was applied to analyze Str and Bru in brain samples collected from rats dosed with SAs (12 mg/kg/day) for 15 days, with or without pretreatment of TGP. Although various analytical methods had been applied to determine Str and Bru in vitro [41,42] or in peripheral systems [43,44], few studies have examined the time-course change and distribution of Str and Bru in the brain. This is the first study to show the absorption and localization of SAs in rat brains.

SAs, a class of dihydroindole-type alkaloids, share similar structural and physicochemical properties [45]. Compared to the chemical structure of Str, Bru just has two additional methoxy groups in its benzene ring. In addition, alkaloids are particularly easy to ionize and become hydrophilic ionic compounds before separation in acidic conditions. Hence, it is a great challenge to achieve total chromatographic separation of Str and Bru using conventional RP-C18 columns. In the present study, a core-shell type ADME column was selected for chromatographic separation following careful comparison and evaluation. The adamantyl-functional group modified stationary phase has appropriate surface polarity (0.65), which is much higher than that of ordinary RP-C_18_ columns (0.4). Therefore, it provided satisfactory retention for moderate hydrophilic compounds [46]. Furthermore, the ADME column has advantages in peak shape and separation efficiency for components with similar structure over other candidates (Figure 7).

The ESI positive ion mode was employed for the structures of SAs with a basic nitrogen atom. Acetaminophen had been successfully used as the IS for LC-MS assays for Str and Bru in a previous experiment conducted by our team [22]. Product ion mass spectra for Str, Bru, and IS were acquired by using injections of individual standard solutions (each at 300.0 ng/mL) to screen precursor and product ion pairs to monitor. The most abundant fragment ions in MRM mode were chosen for the detection of each compound. Determined ion transitions were 335.06→263.70 for Str, 395.17→243.73 for Bru, and 151.94→109.97 for IS.

The absorption and elimination of Str and Bru in the brain were similar (Figure 7) with or without pre-protection of TGP. However, there was a significant difference in the maximal concentration between the SA group and TGP group. Combined with biochemical and histological assessment, it was likely that continuous harmful SA stimuli caused brain injury and neurotoxicity. It also demonstrated that pretreatment with TGP was able to reduce the absorption of Str and Bru into the brain. Based on our findings, it was deduced that the TGP might be able to increase the excretion of toxic substances and lead to neuroprotection. This contributes greatly to TGP’s ability to protect against the neurotoxic effects induced by SAs.

To date, there are no reports concerning the distribution of SAs in the brain. The present study revealed at first that the concentrations of Str and Bru in the hippocampus and cerebellum were higher than in the other brain regions. On the other hand, neurotrophic factors BDNF and NGF decreased after SA exposure in the hippocampus and cerebellum. These findings provide support for the likelihood that the toxic targets of SAs in the CNS may be the hippocampus and cerebellum. Consistent reductions in SA contents were observed in TGP pretreatment rats for the brain distribution study compared with rats dosed only with SAs, which strengthened the likelihood that the TGP could decrease toxic components penetrating into brain. Many factors were likely involved in brain distribution, such as the chemical properties of toxic components and the barrier from membrane transport systems in the brain. Therefore, further investigations are needed to fully reveal the distribution mechanism of SAs in the brain.

Finally, the pre-protective effects of TGP against the neurotoxicity induced by SAs focused on the following two aspects. TGP exerted noticeable pre-protective effects by reducing toxic components in rat brains. In addition, improving the metabolism disorder of endogenous substances increased the resistance to harmful stimuli of toxic components. Paeoniflorin, a monoterpene glucoside, is the most abundant and active ingredient of TGP. It is reported to readily cross the BBB and mainly acts on the CNS [47]. It possesses anti-inflammatory [15], antioxidant, and neuroprotection characteristics [48], all of which have led to greater attention focused on its treatment in neurological diseases. Taken together, TGP showed multiple neural pre-protective effects, mainly thanks to the beneficial properties of paeoniflorin.

## 5. Conclusions

In summary, our in vivo study confirmed that SAs could induce neurotoxicity after continued SA exposure and TGP showed significant pre-protective effects against neurotoxicity induced by SAs by suppressing oxidative stress and reducing the absorption of toxic components. These findings will aid risk assessors when evaluating the potential for adverse effects to humans following exposure to Semen Strychni. Moreover, the neural pre-protective properties of TGP suggested that further preclinical studies aimed at the adjuvant treatment of neurological diseases hold promise.

## Figures and Tables

**Table 1:** List of multiple reaction monitoring parameters for each analyte and internal standard (IS).

Analyte	Collision Energy (eV)	Capillary Voltage (KV)	Cone Voltage (KV)	Structure
Str	30	5	20	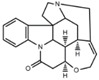
Bru	34	4	15	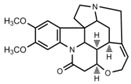
IS	14	5	39	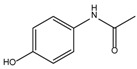

**Table 2:** Results of physical and behavioral testing measured in each group.

	Control Group	SA Group	Low Dose TGP Group	High Dose TGP Group	Positive Group
Body weight	350.8 ± 10.7	279.2 ± 6.1 **	292.9 ± 5.4 ^#^	308.7 ± 11.5 ^#^	276.8 ± 10.1 **
Coat state	1.0 ± 0.7	2.4 ± 0.5 **	2.0 ± 0.7	1.4 ± 0.9 ^#^	2.6 ± 0.5 **
Beam walking test	4.5 ± 0.5	2.5 ± 0.6 **	2.8 ± 0.4	3.6 ± 0.8 ^##^	2.8 ± 0.5 **

** *p* < 0.01 other groups vs. control group; ^#^ *p* < 0.05, ^##^ *p* < 0.01 TGP groups vs. SA group.

**Table 3:** Calibration curves for Str and Bru in brain homogenate (*n* = 3).

Analyte	Liner Range (ng/mL)	Regression Equation	*r*
Str	0.25–200.0	*y* = 6.23 × 10^−2^*x* + 8.97 × 10^−2^	0.9910
Bru	0.05–40.0	*y* = 1.09 × 10^−1^*x* + 5.62 × 10^−2^	0.9905

**Table 4:** Contents of Str and Bru in different brain regions (ng/g).

Region	SA Group		TGP Group	
	Str	Bru	Str	Bru
Cerebral cortex	42.9 ± 3.0	8.4 ± 3.3	38.8 ± 3.2	7.5 ± 1.3
Hypothalamus	31.9 ± 9.5	9.0 ± 1.3	31.2 ± 7.9	7.7 ± 1.7
Striatum	40.9 ± 5.7	9.8 ± 3.9	35.8 ± 6.1	9.5 ± 1.5
Hippocampus	69.6 ± 4.7	28.1 ± 2.7	60.1 ± 1.6 *	19.4 ± 2.3 *
Cerebellum	60.1 ± 2.0	14.7 ± 4.3	45.5 ± 3.1 *	11.5 ± 1.6

* *p* < 0.05 TGP group vs. SA group.

## Supplementary Materials

The following are available online at http://www.mdpi.com/2072-6643/10/4/514/s1, Table S1: The Precision and accuracy of Str and Bru in rat brain homogenate, Table S2: Recovery (*n* = 6) and matrix effect (*n* = 6) of the method.
