# Supplementary material for: Prophylactic Neuroprotection of Total Glucosides of Paeoniae Radix Alba against Semen Strychni-Induced Neurotoxicity in Rats: Suppressing Oxidative Stress and Reducing the Absorption of Toxic Components
Source: Nutrients. 2018 Apr 20;10(4):514. doi: 10.3390/nu10040514 (PMC5946299; doi:10.3390/nu10040514)
Supplement: Supplementary file 1 [file nutrients-10-00514-s001.zip › nutrients-275665-supplementary.pdf]

Selectivity (n = 6) was evaluated by comparing the chromatograms of blank brain homogenate samples, spiked brain homogenate with analytes and IS and brain homogenate obtained 0.5 h after oral administration of SAs to assess whether endogenous or other irrelevant compounds could be observed at the retention time of the analytes. Calibration curves were constructed by plotting the concentration (x) versus analyte-to-IS peak area ratio (y) with seven calibrators using $1/x^2$ as a weighting factor. The intra-day and inter-day precision and accuracy were evaluated by determining each component at three different concentrations (low, medium and high concentrations) of QC samples in six replicates analyzed over three consecutive days. Extraction recovery and matrix effect were evaluated at three QC levels. Three sets of samples were prepared: (A) samples fortified and extracted as described in "sample preparation"; (B) samples fortified with QC solutions and IS solution post-preparation; (C) standard solutions at the same concentrations. Recovery was calculated by dividing the peak area of set A by the peak area of set B, while matrix effect was calculated by dividing the peak area of set A by the peak area of set C. Stability was tested under four different storage conditions: 8 h at room temperature; three freeze-thaw cycles; autosampler condition (4°C) for 12 h and storage at -80 °C for a month. Experiments were prepared in triplicate at three QC concentrations

Table S1: The precision and accuracy of Str and Bru in rat brain homogenate.

Analyte	Concentration (ng/mL)	Intra-day (n = 6)		Inter-day (n = 18)	
		Precision (RSD%)	Accuracy (RE%)	Precision (RSD%)	Accuracy (RE%)
Str	0.25	14.3	16.8	15.8	7.9
	0.50	9.8	-3.8	12.3	0.7
	10.0	6.2	-2.1	5.4	-2.4
	160.0	1.8	1.6	2.0	0.9
Bru	0.05	18.0	11.8	17.8	14.5
	0.1	11.5	10.0	12.9	4.6
	2.0	5.4	-3.6	5.2	-4.7
	32.0	1.5	1.4	4.2	-0.8

Table S2: Recovery (n = 6) and matrix effect (n = 6) of the method.

Analyte	Concentration (ng/mL)	Recovery (mean±SD)	Matrix effect (mean±SD)
Str	0.50	89.8±7.1	96.5±13.2
	10.0	93.5±3.7	99.5±2.6
	160.0	99.4±3.9	98.1±3.5
Bru	0.1	93.4±9.3	98.5±5.2
	2.0	100.8±13.0	103.8±3.5
	32.0	98.1±3.5	97.5±2.3
IS	50.0	98.1±0.8	100.4±2.8
